# Supplementary material for: Human Neutrophils Generate Extracellular Vesicles That Modulate Their Functional Responses
Source: Cells. 2022 Dec 29;12(1):136. doi: 10.3390/cells12010136 (PMC9818892; doi:10.3390/cells12010136)
Supplement: Supplementary file 1 [file cells-12-00136-s001.zip › cells-1986671-supplementary.pdf]

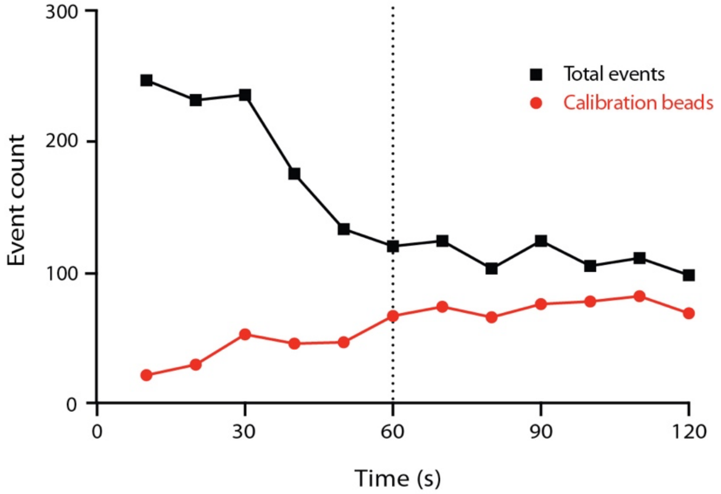

Figure S1. Acquisition of flow cytometry events after fluctuations in numbers had stabilized. Supernatants from fMLP-stimulated neutrophils were centrifuged at 1,000 g; the resulting supernatants were centrifuged at 18,000 g, and the pellets were resuspended and spiked with florescent calibration beads prior to flow cytometry analysis. Detection of total events (black trace) and calibration beads (red trace) was monitored over time and found to stabilize after one minute at constant flow.

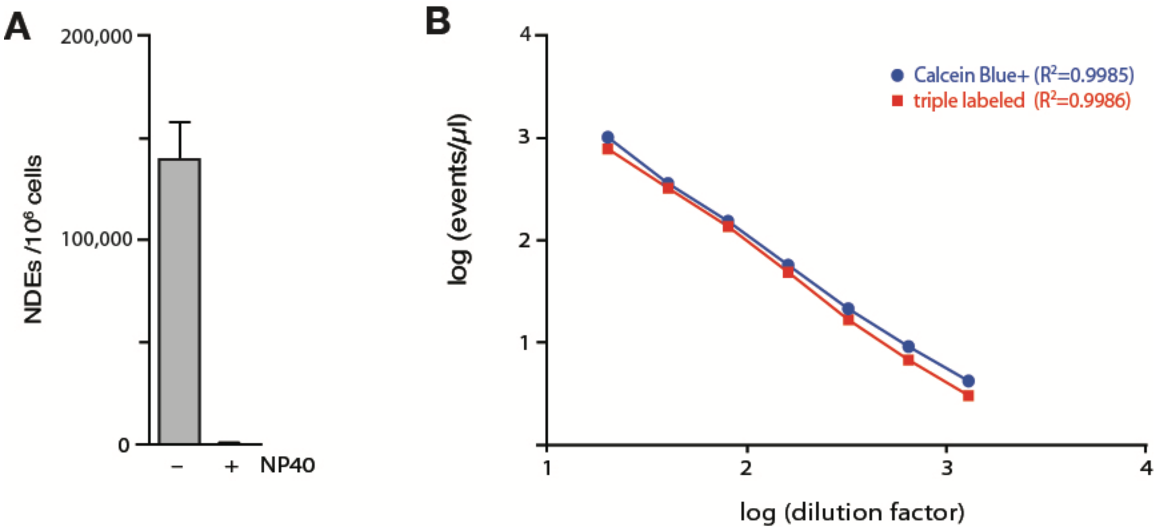

Figure S2. Effect of detergents or of NDE dilution on their detection by flow cytometry. (A) NDEs from the cavitates of fMLP-stimulated neutrophils were loaded with Calcein Blue AM and incubated (1 min, RT, with brief initial vortexing) in the presence or absence of 0.5% NP40 prior to FACS analysis. Mean ± s.e.m. from 3 independent experiments. (B) NDEs from the cavitates of fMLP-stimulated neutrophils were serially diluted prior to flow cytometry re-analysis, monitoring either calcein-positive events only or triple-labeled NDEs (Calcein Blue, CD66b, Annexin-V).

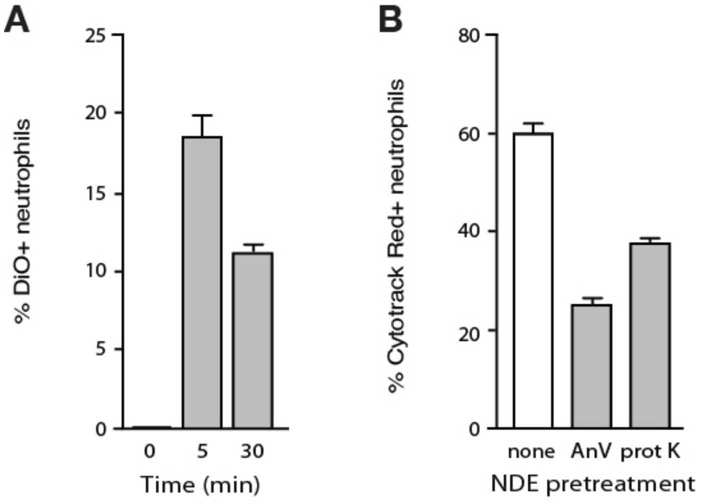

Figure S3. Transfer of NDE fluorescence to autologous neutrophils. (A) NDEs from unstimulated neutrophils disrupted by nitrogen cavitation were stained with the membrane dye, DiO, washed, and co-cultured with autologous neutrophils at a ratio of 5 NDEs per recipient cell. Reactions were stopped at the indicated times, and the cells were collected and analyzed by flow cytometry for DiO fluorescence. (B) NDEs from fMLP-activated neutrophils disrupted by nitrogen cavitation were stained with Cytotrack Red, washed, and treated as follows. NDEs were resuspended in Annexin Binding Buffer and exposed to Annexin V (20 min, 37°C, in the dark), and washed again ("AnV"); or resuspended in HBSS and exposed to proteinase K (20 min, 37°C), and washed again ("prot K"); or resuspended in HBSS, incubated (20 min, 37°C), and washed again ("none"). NDEs were finally co-cultured with autologous neutrophils (5 NDEs per recipient cell, 30 min, 37°C). Reactions were then stopped and the cells were analyzed by flow cytometry for Cytotrack Red fluorescence.

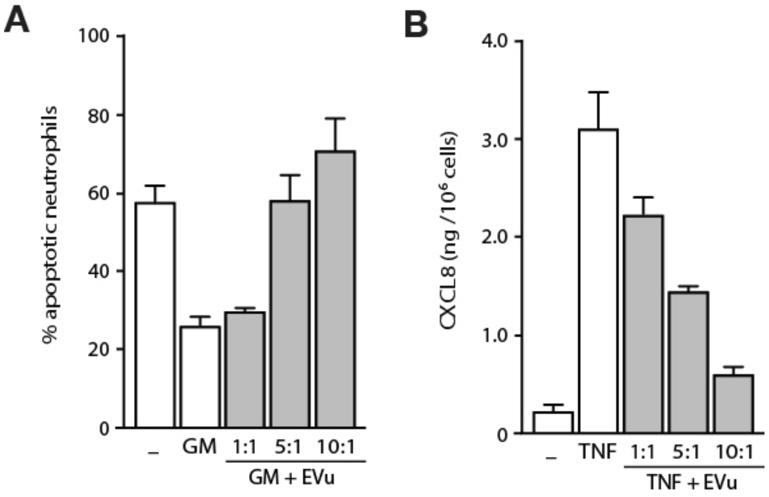

Figure S4. Effect of NDE ratios on human neutrophil responses. (A) Cells were cultured for 18 h in the absence of any stimulus (“–”), in the presence of 1 nM GM-CSF, or with both GM-CSF and NDEs from the cavitates of resting neutrophils (“EVu”) at the depicted NDE:neutrophil ratios. Cells were then processed for flow cytometry analysis of apoptotic cells (AnnexinV+, PI-). (B) Neutrophils were cultured for 6 h in the absence of any stimulus (“–”), in the presence of 100 U/ml TNFα, or with both TNFα and NDEs (“EVu”) at the depicted NDE:neutrophil ratios. Culture supernatants were analyzed by ELISA for their CXCL8 content.

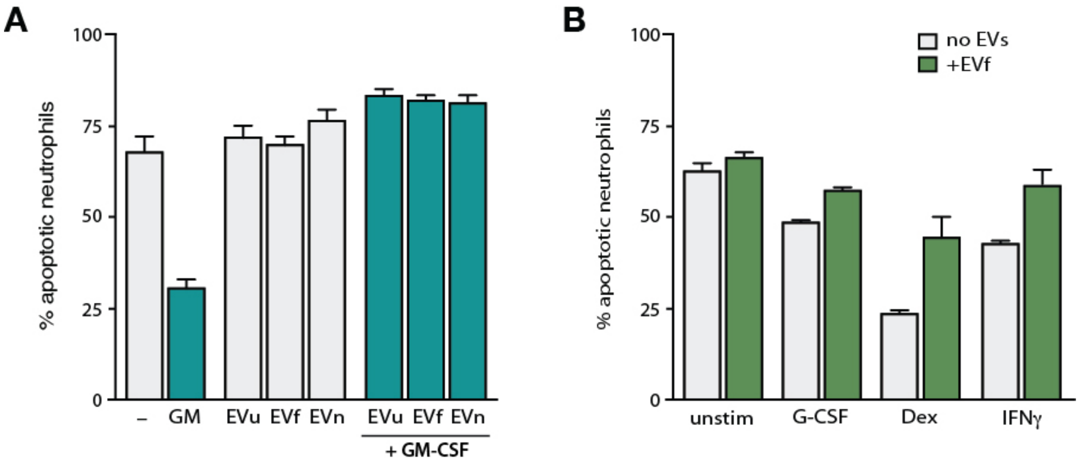

Figure S5. Effect of NDEs from activated cells on autologous neutrophil apoptosis. (A) Neutrophils were cultured for 18 h in the absence of any stimulus (“–”) or in the presence of 1 nM GM-CSF, NDEs from the cavitates of fMLP-activated neutrophils (“EVf”, at a 5:1 NDE:cell ratio), NDEs from the culture supernatants of fMLP- activated neutrophils (“EVn”, at a 5:1 NDE:cell ratio), or a combination thereof. Cells were then processed for flow cytometry analysis of apoptotic cells (AnnexinV+, PI-). This experiment is representative of two. (B) Neutrophils were cultured as described above but stimulated with either 1000 U/ml G-CSF, 100 nM dexamethasone, or 100 U/ml IFNγ.

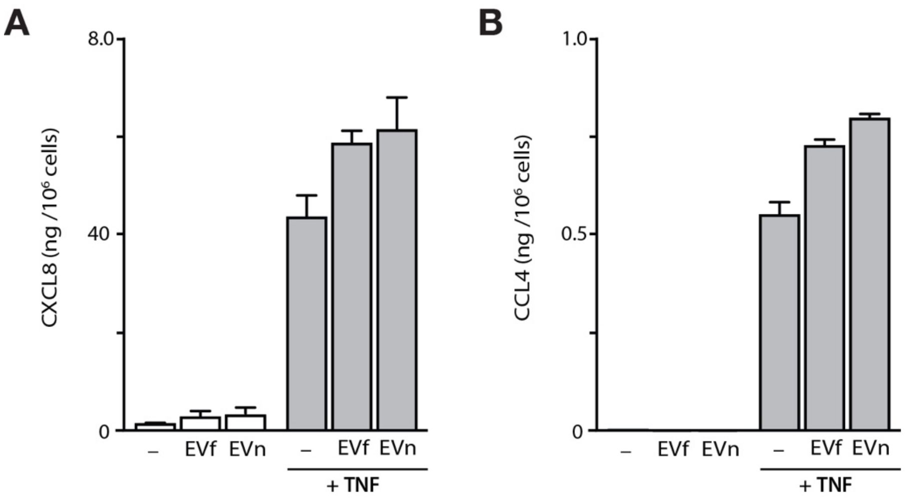

Figure S6. Effect of NDEs from activated cells on autologous neutrophil chemokine secretion. Neutrophils were cultured for 6 h in the absence of any stimulus ("–") or in the presence of 100 U/ml TNFα, NDEs from the cavitates of fMLP-activated neutrophils ("EVf", at a 5:1 NDE:cell ratio), NDEs from the culture supernatants of fMLP- activated neutrophils ("EVn", at a 5:1 NDE:cell ratio), or a combination thereof. Culture supernatants were analyzed by ELISA for their CXCL8 or CCL4 content. Each of the depicted experiments is representative of at least two.

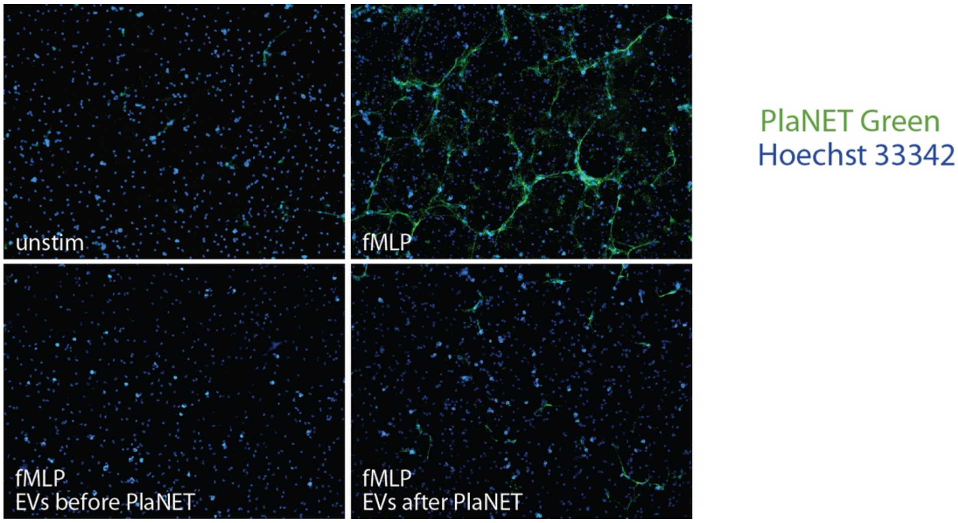

Figure S7. Interference by NDEs with NET detection using PlaNET Green. Neutrophils were cultured for 4 h in the absence of any stimulus ("unstim") or in the presence of 100 nM fMLP. Reactions were stopped by the addition of cold PBS containing 0.5 mM PMSF. At his point NDEs from the cavitates of unstimulated neutrophils were either added before or after the PlaNET reagent, for 15 min on ice at a 5:1 (NDE:cell) ratio. Samples were then fixed in the presence of Hoechst 33342 as described in *Methods* and visualized by epifluorescence microscopy (10X magnification).
